# The Differences in Spatial Luminescence Characteristics between Blue and Green Quantum Wells in Monolithic Semipolar (20-21) LEDs Using SNOM

**DOI:** 10.3390/nano12193386

**Published:** 2022-09-27

**Authors:** Aixing Li, Yufeng Li, Jie Song, Haifeng Yang, Ye Zhang, Peng Hu, Zhenhuan Tian, Minyan Zhang, Qiang Li, Feng Yun

**Affiliations:** 1 Shaanxi Provincial Key Laboratory of Photonics & Information Technology, Xi’an Jiaotong University, Xi’an 710049, China; 2 Solid-State Lighting Engineering Research Center, Xi’an Jiaotong University, Xi’an 710049, China; 3 State Key Laboratory of Transient Optics and Photonics, Xi’an Institute of Optics and Precision Mechanics, Chinese Academy of Sciences, Xi’an 710119, China

**Keywords:** semipolar LEDs, localization states, scanning near-field optical microscopy

## Abstract

The differences in spatially optical properties between blue and green quantum wells (QWs) in a monolithic dual-wavelength semipolar (20-21) structure were investigated by scanning near-field optical microscopy (SNOM). The shortest wavelength for green QWs and the longest wavelength for blue QWs were both discovered in the region with the largest stress. It demonstrated that In composition, compared to stress, plays a negligible role in defining the peak wavelength for blue QWs, while for green QWs, In composition strongly affects the peak wavelength. For green QWs, significant photoluminescence enhancement was observed in the defect-free region, which was not found for blue QWs. Furthermore, the efficiency droop was aggravated in the defect-free region for green QWs but reduced for blue QWs. It indicates that carrier delocalization plays a more important role in the efficiency droop for QWs of good crystalline quality, which is experimentally pointed out for the first time.

## 1. Introduction

GaN-based white light-emitting diodes (LEDs) have the advantages of low power consumption and long life and are regarded as next-generation solid-state lighting sources (SSLs) [1]. Phosphorus-free monolithic blue/green/red multi-wavelength LEDs are one of the most straightforward paths to achieving white LEDs with a high color rendering index (CRI). Achieving high performance at long wavelengths, such as green and yellow light on c-plane polar substrates, remains difficult due to the quantum confinement Stark effect (QCSE) and the confinement of indium doping in GaN [2,3]. On the other hand, the wavelength stability of the semipolar structure was found with increasing pump power density. Quantum wells (QWs) in the semipolar direction have larger wavefunction overlap and shorter carrier lifetimes. Therefore, it is worth investigating monolithic dual-wavelength LEDs grown on semipolar structures, especially the spatial luminescence characteristics differences of different QWs. Such high-efficiency monolithic phosphor-free white LEDs can be used as light sources for high-speed visible-light communication (VLC), high CRI lighting, and other applications.

Reports on dual-wavelength semipolar structures so far have mainly focused on carrier transport and the polarization ratio of dual-wavelength emissions by macroscopic electroluminescence and photoluminescence (PL) techniques [4,5,6,7,8]. Direct observation of spatial luminescence characteristics among dual-wavelength semipolar QWs is difficult due to limited resolution, which is crucial to fully understanding the recombination mechanism in multi-wavelength QWs. In our work, dual-wavelength semipolar (20-21) LEDs containing blue and green QWs are grown. Defect and stress distributions in both QWs are examined. Near-field PL properties, as a function of excitation power density, are measured using scanning near-field optical microscopy (SNOM). The differences in spatial luminescence characteristics, such as PL intensity, peak wavelength, the full width at half maximum (FWHM), and efficiency droop due to the stress and defect distribution, are described, which can provide direct insight into the different recombination mechanisms in multi-wavelength semipolar QWs. 

## 2. Materials and Methods

The (20-21) blue and green dual-wavelength InGaN/GaN LEDs were grown on a (20-21) GaN template that was firstly grown on a c-plane-like sapphire sidewall of a (22-43) patterned sapphire substrate (PSS) by metal–organic chemical vapor deposition (MOCVD). Details of the growth of the (20-21) GaN/sapphire template can be found elsewhere [9,10]. The QW structure consists of a 0.5 µm undoped GaN, a 1.5 µm n-type GaN with a silicon concentration of 1 × 10^19^ cm^−3^, 2 pairs of In_0.22_Ga_0.78_N/n-GaN (2 nm/7 nm) blue QWs, 3 pairs of In_0.27_Ga_0.73_N/GaN (3.5 nm/7 nm) green QWs, an 18 nm GaN barrier, a 150 nm p-type Al_0.15_Ga_0.85_N electron blocking layer (EBL), a 40 nm p-type GaN layer with a Mg concentration of 2 × 10^19^ cm^−3^, and a 10 nm p^+^GaN contact layer with a higher Mg doping concentration.

Near-field PL measurements were performed at room temperature (RT) with a SNOM apparatus (NTEGRA, NT-MDT, Moscow, Russia) operating in illumination mode. PL was excited directly into QWs by a 405 nm continuous wave (CW) laser diode (Power Technology, Alexander, AR, USA) through an aluminum-coated optical fiber probe with an aperture of 100 nm diameter. The PL signal was collected from the polished backside of the sapphire substrate by a 100× inverted objective. After passing a 413 nm long-pass filter, the PL signal was directed to a photomultiplier tube (PMT) for PL mapping. PL spectra were recorded by a diffraction grating spectrometer (HORIBA-iHR550, Jobin Yvon, Kyoto, Japan) with an optical resolution of 0.02 nm. The excitation-dependent near-field PL measurements at different positions were performed from 0.037 to 1.49 MW/cm^2^, and the injected carrier density was estimated from 2.54 × 10^15^ to 1.02 × 10^17^ cm^−^^3^ [11]. 

Micro-Raman spectroscopy measurements were performed at RT using a grating Raman spectrometer (LabRAM HR Evolution, HORIBA Jobin Yvon, Paris, France) with a 532 nm solid-state laser diode as the excitation source. The Raman spectra were measured from the GaN top surface with 700 nm spatial resolution using a 50× objective to focus and collect the scattered laser light. Laser power at the sample was about 50 mW. In order to obtain the Raman mapping, the sample was scanned underneath the laser beam using a motorized XY stage with a scanning resolution of 500 nm. The temperature-dependent macroscopic PL measurements were carried out to evaluate the internal quantum efficiency (IQE) of blue and green QWs. The sample was placed in a closed-cycle mechanical cryogenic system (Optistat Dry BL4, Oxford Instrument, Oxford, UK) with a temperature range from 4 to 300 K and excited with a 405 nm CW laser diode at the excitation power density of 2.23 W/cm^2^.

## 3. Results and Discussion

**Figure 1:**
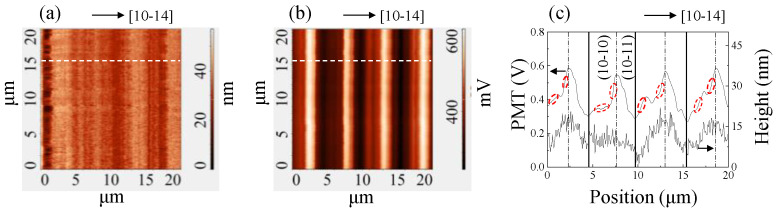
(**a**) AFM image of the p-type GaN surface and (**b**) near-field PL mapping of the (20-21) dual-wavelength LED acquired over a 20 × 20 µm^2^ area; (**c**) surface morphology profiles and PL intensity extracted from the white dashed lines in (**a**,**b**).

AFM image and near-field PL mapping (using spectra integrated intensity) over a 20 × 20 μm^2^ area are shown in Figure 1a,b, respectively. The PL excitation density was about 2.6 MW/cm^2^. In the AFM image, ridge-like structures were observable, which has previously been reported for (20-21) InGaN. Additionally, it has been pointed out that the surface undulations originate from (10-11) and (10-10) microfacets [12]. Figure 1c exhibits the cross-sectional profiles of topography and PL intensity extracted from the white dashed lines in Figure 1a,b; the (10-10) and (10-11) microfacets were separated by a dashed line in each period of undulation. The QW emission intensity shows a strong correlation with the topography. The strongest PL intensity is always found at the highest point of the ridge structures. The difference between the bright and dim regions is about 54.5% of the maximum emission amplitude. The surface undulation was very small, and the average angle of microfacets with respect to [10,11,12,13,14] was calculated to be only 0.2°. Therefore, the excitation power was uniform across the sample. The change in PL intensity does not exactly follow the surface undulation pattern, such as regions marked by red dot circle. The spatial difference of the light extraction efficiency (LEE) from the sapphire side, calculated by the Monte-Carlo ray-tracing method, is only about 10%, which is insufficient to explain the variation in PL intensity (see Figure S1). 

**Figure 2:**
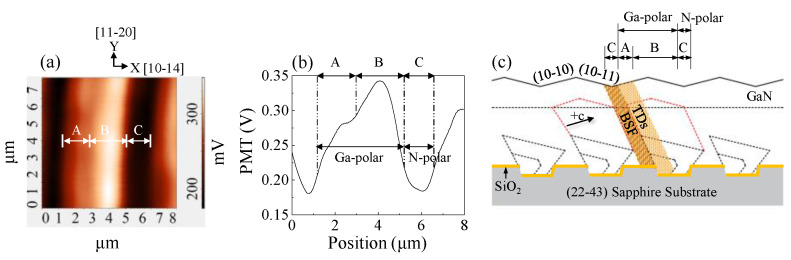
(**a**) An 8 × 8 µm^2^ near-field PL mapping; (**b**) cross-sectional profiles of the near-field PL intensity extracted at y = 3.8 µm in (**a**); (**c**) schematic of (20-21) GaN growth from sapphire (0001) sidewall.

Figure 2a displays the near-field PL mapping over one period of surface undulation under 1.49 MW/cm^2^. Figure 2b shows the cross-sectional profiles extracted at y = 3.8 µm. The total widths of the Ga-polar GaN (regions A and B) and N-polar GaN (region C) were measured as 4.1 and 1.3 μm, respectively. The ratio of the wing widths indicates that the Ga-polar GaN grows approximately twice as fast as the N-polar GaN. Such a growth rate difference is sensitive to growth temperature and is attributed to the differences in adsorption and desorption rates and/or relative chemical stability. The average PL intensity of the B region is 1.2 and 1.6 times higher than that of the A and C regions, respectively. The PL intensity mapping indirectly reflects the distribution of the nonradiative recombination centers (NRCs). Figure 2c presents a schematic of (20-21) GaN growth from the sapphire (0001) sidewall. Threading dislocations (TDs) (region A) were generated in the Ga-polar GaN in the initial stage of the growth due to the lattice mismatch between GaN and sapphire and were propagated to the GaN surface along the [10–10] direction. When the growth mode changed from three-dimension (3D) nucleation to 2D lateral growth, the TDs were bent and merged and eventually stopped from extending upward [13], which resulted in an almost defect-free region B. In region C, stacking faults (SFs) tend to be generated in N-polar GaN. This is often the case with heteroepitaxial semi- or nonpolar GaN growth using PSS or sidewall lateral epitaxy overgrowth [14,15]. Since the etched sidewalls are not exact c-plane sapphire sidewalls in PSS, growth errors can lead to the formation of SFs on adjacent sapphire sidewalls [16,17]. Ga adatoms’ diffusion on the exposed c-plane of GaN acts as a source of SF generation [18]. Under nitrogen-rich conditions, Ga adatoms are easily trapped at zincblende fcc sites rather than wurtzite hcp sites, leading to the formation of SF in N-polar GaN [19]. The heteroepitaxial growth mechanism and defect distribution of (20-21) GaN have been well described in the literature [9,17,20]. SFs and TDs are related to NRC [13,21]; therefore, the emission intensity of regions A and C with SF and TD is lower than that of defect-free region B.

**Figure 3:**
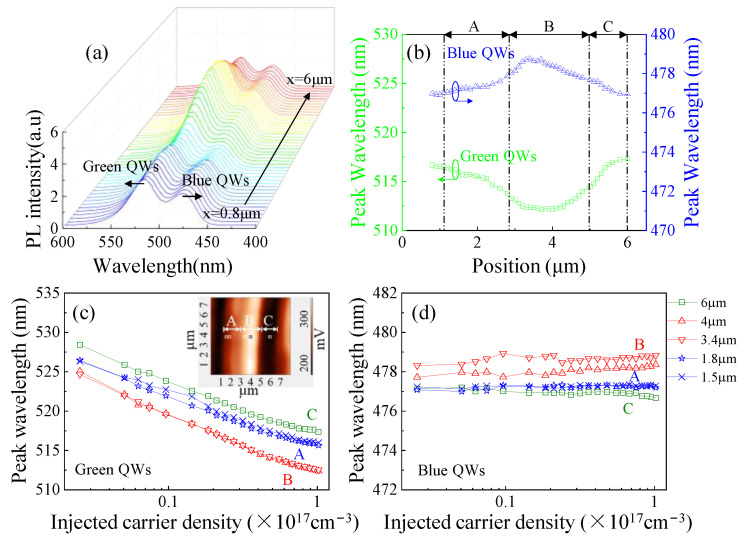
(**a**) Near-field PL spectra collected from different positions in Figure 2a, from x = 0.8 μm to x = 6 μm; (**b**) the peak wavelength of green and blue QWs; excitation-dependent peak wavelength of (**c**) green and (**d**) blue QWs. The inset of Figure 3c shows the corresponding locations of the measurement points in the PL mapping.

Near-field PL spectra were measured at different positions along the [10–14] direction during one period of PL intensity variation, from x = 0.8 to 6 µm, as shown in Figure 3a. The peak wavelength distributions of the green and blue QWs are shown in Figure 3b. The maximum variations of the PL peak wavelength for green and blue QWs were about 5 and 2 nm, which were much smaller than that observed in the (0001) QW (normally 10–20 nm) [22]. It is clear that the (20-21) QWs have a more uniform potential distribution than the (0001) QW. Similar to intensity, the PL peak wavelength shows ridge distribution along the [10–14] direction. Opposite trends along the [10–14] direction were observed in green and blue QWs. Region B possesses the shortest wavelength for green QWs and the longest wavelength for blue QWs. Figure 3c,d present the excitation power density-dependent peak wavelength of green and blue QWs at different spatial locations of A, B, and C, marked in the inset of Figure 3c. As the injected carrier density increases, the peak wavelength of green QWs at all positions (from x = 0.8 to 6 μm) shows blueshifts of 10.5, 12.4, and 11.1 nm in regions A, B and C, respectively. The blue QWs exhibit less blueshift (<1 nm) than green QWs, as shown in Figure 3d. Such blueshift was attributed to the screening of the QCSE by photogenerated excitons and/or carriers [23]. Compared to green QWs, the QCSE in blue QWs is weaker due to smaller internal electric fields induced by lower concentrations of indium [24]. 

**Figure 4:**
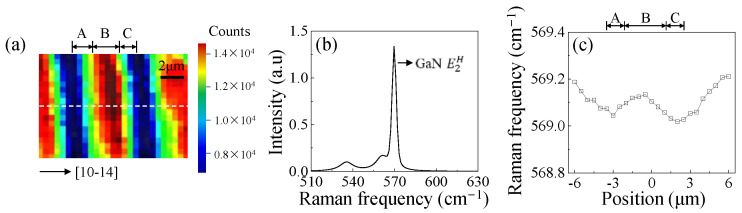
(**a**) GaN $E_{2}^{H}$ mode peak intensity mapping in the p-GaN layer; (**b**) a micro-Raman spectrum extracted from (**a**); (**c**) the GaN $E_{2}^{H}$ peak position variation along the white dashed lines in (**a**).

To investigate the origin of PL peak wavelength distributions of blue and green QWs, micro-Raman measurement was performed on the (20-21) LED. Since the phonon frequency of the $E_{2}^{H}$ Raman peak is sensitive to biaxial stress along the c-plane of GaN, the $E_{2}^{H}$ phonon mode of GaN can be used indirectly to evaluate the stress states of the GaN layer [25]. Figure 4a shows the Raman intensity mapping of the p-GaN layer. The intensity mapping replicated the surface undulations with a period of ~5.4 μm. Stronger Raman intensity was always found in region B compared to regions A and C. Figure 4b displays a Raman spectrum extracted from Figure 4a. A peak at 569 cm^−1^ is attributed to the $E_{2}^{H}$ mode of GaN [26]. Figure 4c shows the GaN $E_{2}^{H}$ peak position variation along the white dashed lines in Figure 4a. The phonon frequency of the GaN $E_{2}^{H}$ Raman peak increases from 569.0 cm^−1^ at position 2 μm to 569.2 cm^−1^ at position 6 μm. In addition, the $E_{2}^{H}$ phonon frequency is 568 cm^−1^ for unstrained bulk GaN, and the increased compressive stress in the GaN layer can lead to high-frequency shifts in the GaN $E_{2}^{H}$ mode [27]. Hence, Raman peaks shift to a higher wavenumber from regions A and C to region B; this indicates larger compressive stress in region B. Since the largest compressive stress was observed in region B, the increased QCSE should be responsible for the longest blue emission wavelength. Such stress reduces In incorporation efficiency and shortens the emission wavelength of green QWs [28]. The largest wavelength shift of green QWs thus also appears in the highest stressed region B due to the largest QCSE [29]. The origin of the highest compressive stress in region B can be attributed to its smallest stress relaxation. During epitaxial growth, stress can be relieved by generating dislocations [30,31]. Higher SF and TD densities in regions A and C may result in higher stress relaxation and smaller residual stress than in region B. We also characterized the FWHM of the Raman peaks and found that the difference between different regions is less than 0.1 cm^−1^; hence, it is not shown here.

**Figure 5:**
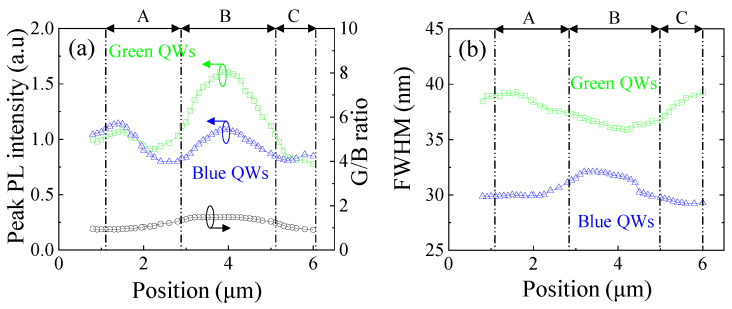
(**a**) The peak PL intensity of green and blue QWs and their peak PL intensity ratios; (**b**) the FWHM of green and blue QWs at different positions.

The near-field PL peak intensity and the green-to-blue intensity ratio are shown in Figure 5a. The PL intensities of blue and green QWs have been normalized to their QW number (2 for blue and 3 for green) for a fair comparison. In region B, green QWs show a significantly enhanced peak PL intensity due to fewer defects than regions A and C, while it is not obvious in blue QWs. Due to higher In incorporation efficiency in regions A and C, more localization states induced by In-rich sites were generated than in region B. These localization states can prevent carriers and/or excitons from being trapped by TDs and SFs, thereby reducing nonradiative recombination. Therefore, there was no obvious PL enhancement in region B for blue QWs. In green QWs, new TDs as NRCs are introduced due to higher In content than in blue QWs, resulting in more NRCs, which can be supported by their lower IQE for green QWs (5.2%) than blue QWs (30.1%) (see Figure S2). Moreover, carrier diffusion length in green QWs is longer than that in blue QWs [22], resulting in localization states in green emissions associated with NRCs [32]. Consequently, the PL intensity of green QWs is significantly reduced in regions A and C, thus leading to the smallest G/B ratio. The spatial PL results in blue and green QWs also verify the role of the carrier localization states around defects and are strongly affected by the crystalline quality. The PL intensity difference of blue and green QWs in Figure 5a is not consistent with their IQE difference. This is due to the fact that the blue QW numbers exceed those of green QWs and the excitation source is closer to the top green QWs, resulting in a higher carrier density being injected into green QWs rather than blue QWs. 

Figure 5b depicts the PL FWHM of blue and green QWs at different positions. The minimum FWHM of green QWs and the maximum FWHM of blue QWs both appear in region B. It is well known that both defect density and QCSE affect the FWHM. In blue QWs, the strongest QSCE in region B leads to the largest FWHM [29]. In green QWs, the lowest defect density in region B results in the smallest FWHM [33,34]. The poorer crystal quality and larger QCSE in green QWs should be responsible for its overall larger FWHM than blue QWs. These results also demonstrate that green QWs are more sensitive to defects than blue QWs.

Excitation-dependent near-field PL measurement was carried out at different positions, and PL external quantum efficiency (*EQE*) was calculated by

(1) $$EQE=k\frac{I_{PL}}{P_{PL}}$$

where $I_{PL}$ and $P_{PL}$ are the PL intensity integrated over the photon energy emitted from QWs and the power of the excitation source, respectively. k is a constant affected by the collection efficiency of the 100× inverted objective, the measurement parameters of PL, light extraction, and absorption efficiency by QWs and does not depend on either excitation power density or measurement position [35,36,37].

**Figure 6:**
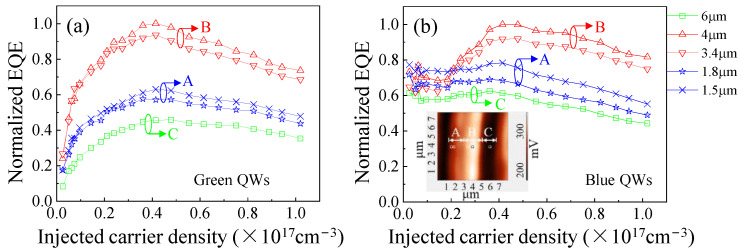
The normalized EQE at different positions of (**a**) green QWs and (**b**) blue QWs, respectively.

Figure 6a,b show the normalized EQE measured from 5 different locations of regions A, B, and C. EQEs of blue and green QWs were separated, calculated, and normalized to the maximum value of all curves. The efficiency droop is calculated as the percentage of efficiency reduction at the injected carrier density used (1.02 × 10^17^ cm^−3^) with respect to its peak efficiency. For green QWs, the efficiency droop is 24.1% in region A, 26.8% in region B, and 22.5% in region C, respectively. Region B has the highest peak efficiency and the largest efficiency droop, which reveals the relevance of defects for droop. It has been reported that carrier leakage at high-level excitation is quantitatively very similar for different defect densities, but high defect densities can suppress the peak efficiency due to much nonradiative recombination at low excitation, thus leading to a relatively small droop [38]. Therefore, regions A and C, with high defect densities, have low peak efficiency and a relatively reduced droop. The efficiency droop at high excitation can be assigned to Auger recombination and carrier leakage [39,40].

The efficiency droops for blue QWs are 29.5%, 18.4%, and 29.1% in regions A, B, and C, respectively. However, unlike the green QWs, the blue QWs in region B exhibit a smaller efficiency droop than those in regions A and C, indicating a different mechanism. Enhanced QCSE in region B would aggravate efficiency droop, which is contrary to our results and is ruled out [41]. At low excitation, the carriers are strongly confined in the localization states, leading to enhanced radiative recombination. When increasing excitation density, the available localization states are saturated gradually and the carrier delocalization will be enhanced, which will enhance the defect-related nonradiative recombination [42,43,44]. Therefore, blue QWs in regions A and C show a comparable quantum efficiency with that in region B at low excitation (<0.3 MW/cm^2^) due to its high carrier localization. At high excitation (>0.6 MW/cm^2^), blue QWs in regions A and C show a faster reduction in quantum efficiency, owing to the delocalization of carriers and high defect density. The difference in the spatial-resolved efficiency droops between blue and green QWs demonstrates that carrier delocalization plays a more important role in efficiency droops for QWs with good crystalline quality.

The EQE of green QWs in regions A and C increased at a higher rate than blue QWs in the initial excitation. One reason could be that green QWs are more sensitive to defects. We fitted the EQE curves of blue and green QWs in regions A and C using the ABC model [45]. The nonradiative and Auger coefficients for green QWs were: A = 5.5 × 10^5^ s^−1^, C = 4 × 10^−28^ cm^−3^s^−1^ (region A); A = 4.5 × 10^5^ s^−1^, C = 2 × 10^−28^ cm^−3^s^−1^ (region C). The coefficients for blue QWs were: A = 3.5×10^5^ s^−1^, C = 5 × 10^−28^ cm^−3^s^−1^ (region A); A = 4 × 10^5^ s^−1^, C = 4.7 × 10^−28^ cm^−3^s^−1^ (region C). At low excitation, defect-related nonradiative recombination is the main recombination mechanism, and thus, a larger nonradiative coefficient is observed in regions A and C for green QWs.

## 4. Conclusions

In summary, we used SNOM to study the difference in spatial luminescence characteristics between blue and green QWs in monolithic semipolar (20-21) LEDs and analyzed their correlation with defect and stress distributions. The peak wavelengths of blue and green QWs present a ridged distribution with the opposite trend. It is suggested that for short-wavelength blue semipolar QWs, the peak wavelength is more easily affected by the stress instead of the In composition. The smallest PL intensity ratio of green to blue light is displayed in the defective region, indicating that green QWs is more sensitive to defects than blue QWs due to higher defect densities and longer recombination lifetimes. Additionally, for green QWs, the efficiency droops in the defective regions, which are due to the suppression of peak efficiency by defects, are reduced compared with those in the defect-free area. In contrast, for blue QWs, the efficiency droops in the defective regions are more severe due to density-activated defect recombination by carrier delocalization. This experimentally points out that carrier delocalization plays a more important role in the efficiency droop for QWs with good crystalline quality. It helps researchers to address the EQE droop and “green gap” more clearly, thus facilitating the application of high-efficiency white LEDs in solid-state lighting, visible-light communication, and micro-display technologies.

## Data Availability

Data underlying the results presented in this paper are not publicly available at this time but may be obtained from the authors upon reasonable request.

## Supplementary Materials

The following supporting information can be downloaded at: https://www.mdpi.com/article/10.3390/nano12193386/s1. Figure S1. (a) Simulated LEE map from sapphire side and corresponding depth profile of stripe structure (marked in dark solid line); (b) cross-sectional LEE distribution along the dark dashed line in (a); Figure S2. The temperature dependence of the integrated PL intensity for estimating IQE. The integrated PL intensity has been normalized to 1.0 at 4 K.
