# Supplementary material for: The Differences in Spatial Luminescence Characteristics between Blue and Green Quantum Wells in Monolithic Semipolar (20-21) LEDs Using SNOM
Source: Nanomaterials (Basel). 2022 Sep 27;12(19):3386. doi: 10.3390/nano12193386 (PMC9565590; doi:10.3390/nano12193386)
Supplement: Supplementary file 1 [file nanomaterials-12-03386-s001.zip › nanomaterials-1909603-supplementary.pdf]

nanomaterials

Supplementary Materials

The Differences in Spatial Luminescence Characteristics between Blue and Green Quantum Wells in Monolithic Semipolar (20-21) LEDs Using SNOM

Aixing Li [1,2], Yufeng Li [1,2,*], Jie Song [3,*], Haifeng Yang [1,2], Ye Zhang [1,2], Peng Hu [1,2], Zhenhuan Tian [1,2], Minyan Zhang [1,2], Qiang Li [1,2] and Feng Yun [1,2,*]

1 Shaanxi Provincial Key Laboratory of Photonics & Information Technology, Xi'an Jiaotong University, Xi'an 710049, China
2 Solid-State Lighting Engineering Research Center, Xi'an Jiaotong University, Xi'an 710049, China
3 State Key Laboratory of Transient Optics and Photonics, Xi'an Institute of Optics and Precision Mechanics, Chinese Academy of Sciences, Xi'an 710119, China
* Correspondence: yufengli@mail.xjtu.edu.cn (Y.L.); songjie@opt.ac.cn (J.S.); fyun2010@mail.xjtu.edu.cn (F.Y.)

Section 1: Light Extraction Efficiency Analysis of Sapphire Side

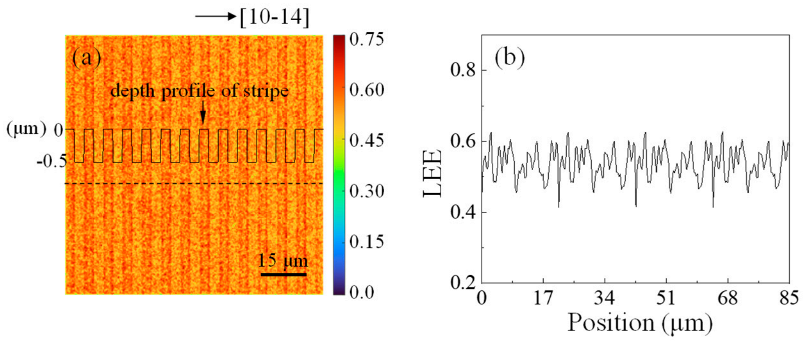

Figure S1. (**a**) Simulated LEE map from sapphire side and corresponding depth profile of stripe structure (marked in dark solid line); (**b**) cross-sectional LEE distribution along the dark dashed line in (**a**).

To estimate the reason for the periodic SNOM-PL intensity mapping, the Monte-Carlo ray-tracing method was used to calculate the LEE distribution of (20-21) LED. In order to simplify the simulation, only three layers were considered, including GaN layer, SiO_2 layer and sapphire substrate. The thickness of GaN film, SiO_2 layer, and sapphire substrate was 5, 0.1, and 20 μm, respectively. The refractive index for GaN, SiO_2, sapphire was set as 2.42, 1.47, and 1.78, respectively. The (20-21) LED model contained a 14 periodic stripe structure embedded in sapphire substrate. The depth, width and period of stripe array were set as 0.5, 3, and ~6 μm, to be consistent with the actual size. The simulation results of LEE map from sapphire side are shown in Figure S1(a), and the corresponding depth profile of stripe structure is marked in a dark solid line. Figure S1(b) depicts the cross-sectional distribution along the dark dashed line in (b). It can be seen that, the LEE exhibits a periodic variation distribution along the [10-14] direction due to the periodic patterned sapphire substrate and SiO_2 layer. However, the difference of two parts is only ~10%, which was insufficient to explain the variation in PL intensity.

Section 2: Internal Quantum Efficiency Analysis of Green and Blue QWs

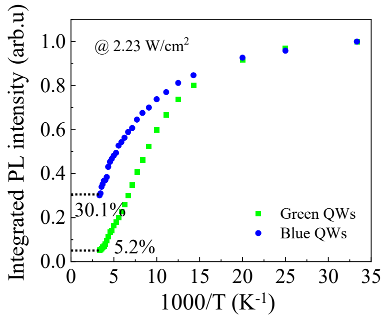

Figure S2. The temperature dependence of the integrated PL intensity for estimating IQE. The integrated PL intensity has been normalized to 1.0 at 4 K.

The temperature-dependent macroscopic PL measurements were carried out to calculate the internal quantum efficiency (IQE), which can reflect the crystalline quality of QWs. The sample was placed in a closed-cycle mechanical cryogenic system (Optistat Dry BL4) with temperature range from 4 to 300 K and excited with a 405 nm CW laser diode at the excitation power density of 2.23 W/cm^2. Figure S2 shows the temperature dependence of the integrated PL intensity as an inverse function of temperature. As the temperature increases, the integrated PL intensity degrades for both QWs due to the activation of nonradiative recombination process and the green QWs decay faster than blue QWs, indicating higher defect density in green QWs. Assuming that the nonradiative recombination is inactive at cryogenic temperatures (typically 4-10 K) and the IQE is approximately 100%, the IQE at 300 K can be defined as the ratio of the integrated PL intensity at 300 K to the integrated intensity at 4 K. Therefore, we can obtain that the IQE is 5.2% for green QWs and 30.1% for blue QWs.
